# Supplementary material for: Are carbon emissions trading and green financial instruments synergistic? -Comprehensive quantitative research based on content analysis
Source: PLoS One. 2024 Mar 7;19(3):e0298601. doi: 10.1371/journal.pone.0298601 (PMC10919601; doi:10.1371/journal.pone.0298601)
Supplement: S1 Data — (ZIP) [file pone.0298601.s001.zip › Data/地级市Z_F工作报告.zip!/░▓╗╒/╗┤▒▒╩╨/└┤╫╘╣1⁄2╓┌║┼í╛┬φ┐╦╩2╛▌═°í┐_2011.docx]

各位代表:现在，我代表市人民政府，向大会报告政府工作，请予审议，并请市政协委员和其他列席人员提出意见。

    一、积极转型，“十一五”发展迈上新台阶刚刚过去的2010年，是我市扩大应对金融危机冲击成果，巩固经济回升向好势头的一年。在市委的坚强领导下，在市人大、市政协的监督支持下，市政府团结和带领全市人民，深入贯彻落实科学发展观，紧紧围绕推进城市转型发展，凝心聚力，攻坚克难，全面完成了市十四届人大三次会议确定的目标任务。预计全年完成地区生产总值455亿元，比上年增长13.5%；财政收入70亿元，增长34.1%；城镇居民人均可支配收入15100元，增长10%；农民人均纯收入5050元，增长11%。

    一年来，我们主要做了以下工作：一是把保持经济增长作为转型发展的首要任务。坚持工业强市主战略，深入开展服务企业年活动，扎实推进中小企业成长工程，规模以上工业增加值增长19%，拉动地区生产总值增长近10个百分点，新增规模企业145家，工业支柱地位更加突出。认真落实强农惠农政策，粮食总产124.6万吨，再创历史新高，规模化养殖比重达70%以上。新增省级农业产业化龙头企业11家，规模以上食品工业产值增长47%。扎实推进重点商贸物流项目建设，凤凰山农贸城、大润发、金鹰国际、徽香昱原早餐工程投入运营。全年社会消费品零售总额126亿元，增长19.2%。完成进出口总额1.7亿美元，增长41.7%。

    二是把扩大有效投入作为转型发展的关键举措。持续加大项目对接和资金争取力度，积极推进政银企合作，全年争取国债和专项资金25亿元，年末银行业金融机构贷款余额309.2亿元，增长27.6%。强化项目带动，落实市级领导联系重点项目责任制，完成全社会固定资产投资378亿元，增长35%。杨柳、青东矿井实现联合试运转，天象龙盟环保纸项目试生产，年产8万吨粗苯加氢精制项目正在调试设备，总投资50亿元的大唐淮北发电厂虎山工程开工建设。国道311和省道303淮北段改建工程竣工通车，泗许高速公路淮北段加快建设。全年谋划重大项目82个，总投资646.4亿元，24个项目进入省“十二五”规划项目库。

    三是把推进城乡统筹作为转型发展的重中之重。城乡一体化综合配套改革试点扩大到“一区一办八镇”，实施重点项目230个，完成投资73.6亿元。编制完成市村庄布点规划，开工建设农民集中居住区37个。开展“清洁乡村、美化家园”活动，扎实推进新农村建设“千村百镇示范工程”，省级示范村扩大到55个。全市305个行政村全部通客车。

   “阳光村务工程”建设扎实有力，开放式村部建设工作被评为全国基层党建创新最佳案例。加强农田水利基本建设，新增和改善灌溉面积18万亩，北淝河洼地治理全面完成。加快土地承包经营权流转，实施农村土地整治和城乡建设用地增减挂钩试点，全年新增规模土地流转面积3.6万亩，整治空心村44个，新增耕地1.4万亩，近万名农村劳动力实现就近就业。

    四是把完善城市功能作为转型发展的有效抓手。强力推进城市东进南扩，全年投资19.5亿元，实施项目27个，新建道路123公里，新东外环、沱河东路、合徐高速淮北连接线建成通车。市人民医院病房大楼投入使用，市图书馆建成，淮北火车站改扩建工程开工建设。持续推进山水生态城市建设，水网一期工程竣工验收，东部山场绿化二期工程全面完成，相山公园被评为国家4A级旅游景区，宇能垃圾发电厂投入运营。认真落实拆迁安置政策，全年完成拆迁面积280万平方米，32个城中村改造项目启动建设。深入推进文明城市创建，在全国117个城市公共文明指数和未成年人思想道德测评中，我市分别位居地级城市第33位和第27位，为争创全国文明城市奠定了坚实基础。

    五是把深化改革开放作为转型发展的动力源泉。突出产业招商和以商招商，成功举办2010中国（淮北）煤矿机械博览会，全年引进市外资金280.9亿元，增长49%；实际利用外资1.8亿美元，增长35%。湖南唐人神、台湾唯一集团、沃尔玛、苏宁电器等一批战略投资者入驻淮北。持续提升园区承载能力，全市开发区完成基础设施投资8亿元，临涣工业园、烈山经济开发区获准筹建省级开发区。鼓励和促进民间投资，全年新增私营企业1597家、个体工商户10096户。积极推进关键环节和重点领域改革，基层医药卫生体制改革、文化体制改革基本完成，扩权强镇试点扎实推进，事业单位绩效工资改革有序进行。濉溪县进入省重点扶持发展的皖北“三市七县”之列，市辖三区一县被纳入省主体功能区规划重点开发区域。

    六是把加强社会建设作为转型发展的内在要求。坚持把更多的资金向民生领域倾斜，当年投入13.1亿元，全面完成33项民生工程。坚持以创业促就业，新增城镇就业4.2万人，城镇登记失业率控制在3.9%，新建农民工创业园10个。完成校舍安全工程17.5万平方米，淮北煤师院更名为淮北师范大学。继市人民医院之后，淮北矿工医院晋升三级甲等医院。建设经济适用房61.4万平方米、廉租房85万平方米，6736户居民搬进新居。体育事业加快发展，王贵贤再获女子散打世界冠军，我市在省第五届残运会上获得的金牌和奖牌总数均名列第二。成功举办建市50周年系列庆典活动，央视《电影之歌》走进淮北，城市知名度不断提升。扎实开展安全生产“打非治违”专项行动，高度重视食品安全监管，组织实施车辆超限超载专项治理，积极做好护校护园安保工作，信访维稳工作走在全省前列，荣获全省社会治安综合治理先进市称号。“五五”普法任务圆满完成。人口和计划生育工作完成省下达目标。第六次全国人口普查取得阶段性成果。民族团结、宗教和睦局面得到巩固。国防动员、人防和双拥优抚工作取得新成绩。公共机构节能成效明显。审计、机关事务管理、档案、外事、对台、地方志、保密工作不断加强，地震、气象、防灾减灾工作扎实推进，妇女、儿童、老龄、残疾人事业实现新发展。

    在过去的一年里，我们扎实开展“创先争优”活动，不断加强政府自身建设。坚持依法行政，自觉接受人大法律监督和政协民主监督，不断密切与民主党派、工青妇等人民团体的联系，全年办理人大代表议案、建议和政协委员提案301件，满意率98%。认真落实廉洁从政的各项规定，政务环境不断优化，政风建设取得新成效。

    各位代表！2010年各项目标任务的顺利完成，为“十一五”划上了圆满句号。回顾过去，我们感到成绩来之不易，经验弥足珍贵。

    这五年，是经济持续快速发展、综合实力大幅提升的五年。全市地区生产总值由205.1亿元增加到455亿元，连跨两个百亿元台阶，年均增长12%；人均地区生产总值达到3300美元，标志着我市经济发展跨入新阶段。财政收入由27.3亿元增加到70亿元，年均增长20.7%。县区经济快速发展，生产总值平均增速17.5%，占全市的比重由42.3%提升到53.8%，市、县、区互动发展的格局逐步形成。

　　这五年，是经济结构不断优化、转型发展步伐加快的五年。大力实施工业振兴计划，规模以上工业企业净增546家，总数达到657家；销售收入超亿元企业83家；工业化率由51.2%提高到60%。食品工业跃升为第二大产业，非煤产业占工业总产值的比重达到48%，五年提高28.5个百分点。我市被列为国家资源枯竭型城市，累计获得国家扶持资金7.56亿元。

　　这五年，是投资力度持续加大、发展后劲明显增强的五年。累计完成固定资产投资1124.5亿元，是“十五”时期的4.2倍，年均增长35.7%，为建市以来投资增速最快的时期。临涣煤焦化一期、孙疃煤矿、五沟煤矿、口子工业园一期、宝迪食品等一批大项目相继投产，科宝生化、金蟾制药、中瑞生物质能源等高新技术企业加快成长，临涣工业园启动建设，一批新的经济增长点加速形成。

　　这五年，是城乡统筹协调发展、市容乡貌变化巨大的五年。全市城镇化率由51.8%提高到58%，城乡一体化进程加快。市财政累计投入“三农”资金30亿元。城乡规划编制基本实现全覆盖，农村公路村村通工程提前一年实现“十一五”目标，广播电视综合覆盖率达到98%，华家湖等一批重点水利工程发挥效益，41.8万农村人口饮水安全问题得到解决。加快推进新区建设和老城区改造，积极建设山水生态城市，节能减排目标全面完成。先后荣获国家园林城市、全省文明城市称号，三次蝉联全国创建文明城市工作先进城市。

　　这五年，是改革开放全面深化、动力活力持续迸发的五年。市属国有企业改制全面完成，新一轮政府机构改革基本结束，106家经营服务性事业单位完成转企改制，义务教育经费保障机制改革不断深化，基层医药卫生体制综合改革走在全省前列。对外开放成效显著，累计利用市外资金769.5亿元，引进亿元以上项目65个，3家世界500强、8家国内500强企业进驻淮北。

　　这五年，是人民生活大幅改善、社会事业全面进步的五年。城镇居民人均可支配收入由8603元增加到15100元，农民人均纯收入由2665元增加到5050元，年均分别增长11.9%和13.6%。

　　五年累计投入民生工程资金28.8亿元，人均受益1674元，多层次、普惠型的民生保障体系基本建立。城镇新增就业16万人，失地农民全部纳入养老保险范围，城镇基本医疗、新型农村合作医疗覆盖城乡。财政预算内科技、教育、文化、卫生、体育事业累计投入61亿元，是“十五”时期的3.4倍。我市成为国家知识产权试点城市，高考本科万人达线率稳居全省第一方阵，村级标准化卫生室覆盖全市，博物馆、体育场等一批标志性文化体育设施投入使用，特奥工作获全国表彰。社会治安连续多年被评为全省先进，人民群众安全感和幸福感明显提升。

　　回顾“十一五”的奋斗历程，我们深切体会到，保持经济社会又好又快发展，推进城市又快又好转型，必须坚持解放思想，开拓创新，把中央和省里的政策与淮北实际相结合，创造性地开展工作；必须坚持改革开放，转型发展，以增量带存量，以结构的优化带动总量的扩张，努力实现速度、质量、效益相统一；必须坚持以人为本，民生为先，着力解决群众最关心、最直接、最现实的利益问题，促进经济社会全面协调可持续性发展；必须坚持依法行政，真抓实干，凝聚方方面面力量，调动社会各界积极性，形成干事创业的良好局面。

　　各位代表！“十一五”取得的成就，在淮北50年发展史上书写了光辉一页，为加快转型、加快发展奠定了坚实基础。这是省委、省政府和市委正确领导的结果，是全市人民团结拼搏、开拓奋进的结果。在此，我代表市人民政府，向全市各族人民、解放军和武警驻淮部队、公安政法干警，向各民主党派、各人民团体和社会各界人士，向所有关心、支持淮北建设与发展的各界朋友，表示衷心的感谢并致以崇高的敬意！在充分肯定成绩的同时，我们也清醒地看到，前进的道路上还存在不少困难和问题。主要表现在：全市经济总量不大，规模企业不多，非公经济发展不快，自主创新能力不强，经济结构调整、城市转型发展刚刚起步。资源环境约束加剧，土地和资金供需矛盾突出，农民持续增收压力较大，统筹城乡改革发展亟待突破，化解社会矛盾、维护社会稳定的任务依然繁重。政府职能转变还不到位，少数干部责任意识不强，工作方法简单，不同程度地存在着官僚主义、形式主义现象，领导科学发展的能力还需进一步提升。对此，我们一定高度重视，采取切实措施，认真加以解决，绝不辜负全市人民的期盼和重托。

　　二、全面转型，谱写“十二五”崛起新篇章未来五年，是我市全面转型的加速期，加快发展的关键期，更是大有可为的黄金发展期。国家积极推动经济布局从沿海向内陆延伸，全面实施促进中部崛起规划，积极支持资源枯竭地区转型发展；省委、省政府把加快皖北发展作为战略重点持续给力，对我市在皖北率先崛起寄予厚望。经过多年的发展和积累，我市人均地区生产总值突破3000美元，进入了工业化城镇化加速扩张期，经济增长的内生动力持续释放，提速效应显著增强。特别是全市上下团结一心、精神振奋，全面转型已成共识，奋力崛起愿望强烈。站在“十二五”新的历史起点，面对竞争激烈、竞相发展的新形势，肩负加快转型、加快发展的新使命，我们将牢固树立不进则退、慢进也是退的进取意识，始终保持昂扬向上、奋发有为的精神状态，大力营造敢干、快干、实干的创业氛围，解放思想破难题、抢抓机遇不懈怠、直面挑战勇担当，努力实现又好又快发展和又快又好转型，奋力争当皖北崛起排头兵。

　　“十二五”时期，全市经济社会发展的指导思想是：坚持以邓小平理论和“三个代表”重要思想为指导，深入贯彻落实科学发展观，大力实施“2256”工程，进一步优化产业结构，加快转变发展方式，协调推进工业化、城镇化、城乡一体化，全面提升城市综合竞争力，努力把我市建成经济实力强、生态环境美、幸福指数优、文明程度高的现代化新型工业城市。指导思想的核心要求是全面落实“2256”工程，即围绕争当皖北崛起排头兵、争创全国文明城市“两争”目标，突出加快转型、加快发展“两快”主题，实现地区生产总值、财政收入、固定资产投入、城镇居民人均可支配收入、农民人均纯收入“五个翻番”；力争地区生产总值增速、财政收入增速、城镇居民人均可支配收入增速、农民人均纯收入增速、工业化率、城镇化率皖北地区“六个领先”。

　　“十二五”时期，全市经济社会发展的主要目标是：综合实力跃上新台阶。地区生产总值突破1000亿元大关。财政收入确保180亿元，力争达到200亿元。

　　全社会固定资产投资累计完成3000亿元以上。规模以上工业增加值突破700亿元，力争达到800亿元。

　　结构调整取得新成效。三次产业比例调整为5:2:33。非煤产业占规模工业总产值的比重达到60%以上，非公经济占全市经济总量的比重达到60%以上，高新技术产业增加值占工业增加值的比重达到30%以上。

　　城乡建设展现新面貌。城市建成区面积达到85平方公里，常住人口85万人，城镇化率达到65%。城乡一体化综合配套改革取得积极成效，建成高标准新型农民社区100个，基本实现城乡基本公共服务均等化。

　　生态环境得到新改善。万元生产总值能耗和化学需氧量、二氧化硫等主要污染物排放总量与下降幅度，控制在省下达目标范围之内。城市污水集中处理率、生活垃圾无害化处理率分别达到90%、75%以上，空气质量二级及以上天数达到95%以上。

　　人民生活达到新水平。城镇居民人均可支配收入突破3万元，农民人均纯收入突破1万元。城镇登记失业率控制在4.2%以内。社会保障基本实现全覆盖。人口自然增长率控制在8.9‰以内。

　　实施“十二五”规划，我们将重点推进“四个发展”：（一）优化经济结构，提升产业层级，推进转型发展。

　　一是加速工业振兴崛起。牢固树立工业是立市之本、强市之基的理念，大力推进煤电化一体化发展，改造提升食品工业、机械制造、纺织服装等优势产业，打造特色产业集群。抓好资源节约与生态环境保护，大力发展循环经济，积极推行清洁生产，切实转变经济发展方式。到“十二五”末，累计完成工业投资2000亿元，实现产值2000亿元，培育规模工业企业1000家，年产值超百亿元产业8个，形成煤电化、非煤产业两个千亿板块。

　　二是培育发展新兴产业。着眼抢占未来发展的制高点，加强政策支持和规划引导，强化核心技术研发，推进信息化与工业化深度融合，加快培育2-3个先导性、支柱性、战略性新兴产业。着力培育新材料产业，高度重视发展新能源产业，支持中润生物产业化研发，积极发展多晶硅棒及延伸产品，大力发展绿色环保节能产业。到“十二五”末，新兴产业产值突破500亿元。

　　三是壮大提升服务业。大力发展现代金融服务业，积极引进股份制商业银行在淮北设立分支机构，加快发展小额贷款公司和担保公司，发展村镇银行，改造农村信用社，重视引进保险、证券公司等金融机构；积极发展现代物流业，建设烈山、临涣、百善三大物流园区，南坪、龙湖、凤凰山、段园四大物流基地和专业化物流配送中心，加快建设中国矿业设备博览城。进一步发展消费性服务业，优化城市商业网点空间布局，打造城区湿地公园和隋唐大运河文化旅游两大品牌，支持洪庄文化创意产业园建设，有序发展房地产业，大力发展会展经济，快速发展社区养老事业。

　　“十二五”期间，服务业增加值年均增长12%。

　　（二）持续加大投入，加快项目建设，推进率先发展。

　　一是推进项目建设。强化项目第一支撑意识，优化投资结构，提高投资效益。重点推进平山电厂、临涣煤焦化二期、宝迪食品等优势主导产业项目，加快建设东磁新能源、濉溪铝业基地等新兴产业项目。引导投资向民生、“三农”和社会事业等领域倾斜，积极推动现代农业项目和重大基础设施项目建设。

　　“十二五”期间，全社会固定资产投资年均增速达到20%。强化项目谋划生成机制，善做无中生有的文章，谋划和争取一批高、大、新、长的项目，力争在更高层次上快速崛起。

　　二是搭建投资平台。强化园区第一载体意识，加快园区建设开发力度，持续提升园区承载能力，把开发区和工业园区建设成为承接产业转移的主阵地，拉动经济发展的增长极。进一步明确开发区和园区的产业定位及发展方向，推动园区分工合作，错位发展。“十二五”末，全市园区建成区面积突破150平方公里，市开发区力争升级为国家级开发区。

　　三是优化发展环境。强化环境第一要素意识，创新体制机制，提升服务效能。以更大的气魄、更加务实的举措深化经济体制改革，最大限度地激发创新创业热情。完善行政审批制度，推进政企分开、政资分开，政府职能向更加关注社会管理、公共服务和环境保护转变。落实帮扶企业的各项措施，推行为企服务全程代理制，持续优化发展环境。

　　（三）加速新型城镇化，加快城乡一体化，推进统筹发展。

　　一是加速推进新型城镇化。加快城市“东进、西延、南扩”，实现相山组团与濉溪组团、烈山组团基础设施共建共享，拓展城市发展空间。加大市域南部开发力度，壮大百善、临涣区域副中心，打造段园、石台、南坪、五沟特色重点镇，优化城市空间布局。推进老城区路网、街道景观整治改造，优化老城区教育、文化等综合服务功能，完善基础设施和公共服务设施，提升城市承载能力。加快东部新区开发建设，“十二五”末，东部新区建成区面积达到35平方公里，人口达到37万人。加快农民市民化步伐，力争今后五年农民变市民人数超过10万人。

　　二是加快推进城乡一体化。扎实开展城乡一体化综合配套改革试点，加快集中居住区建设，在全省率先形成城乡一体化发展格局。大力发展现代农业，调整农业结构，提升农业综合生产能力，推进农业产业化经营，多渠道增加农民收入。加强农村基础设施建设，继续实施农田排灌工程，全市200万亩农田基本实现旱涝保收。建设农村公路827公里，基本实现主要镇区20分钟上高速公路，主城区至各乡镇1小时内到达。新建一批输变电工程，基本满足城乡用电负荷增长要求。

　　（四）保障和改善民生，加强社会建设，推进和谐发展。

　　一是发展成果城乡共享。大力推动全民创业、自主创业，创造条件增加群众财产性收入和工资性收入。实施更加积极的就业政策，加强职业培训，完善就业援助制度，“十二五”期间，新增城镇就业18万人以上。完善城乡社会保障和社会救助体系，健全城镇职工和居民养老保险制度，实现新型农村养老保险和城乡社会救助全覆盖。加大保障性安居工程建设力度，加快棚户区改造，发展公共租赁房，增加中低收入者住房供给。

　　二是加快发展社会事业。推进义务教育均衡发展，缩小校际差距、城乡教育差距，实现县（区）域内初步均衡。重视学前教育，支持民办教育，关心特殊教育，发展职业教育。全面实施《淮北市中长期人才发展规划纲要（2010-2020年）》，重点推进人才开发10大工程，统筹推进各类人才队伍建设。实施文化惠民工程和文化精品建设工程，推动文化大发展大繁荣。深化医药卫生体制改革，健全覆盖城乡居民的基本医疗保障制度，适时推进公立医院改革。

　　三是建设生态文明城市。坚持绿色发展、低碳发展，全面完成节能减排任务，加快建设资源节约型和环境友好型社会。推动实施淮水北调工程，抓好地下水资源保护利用，实施采煤沉陷区水生态系统综合治理，加快荒山绿化步伐和农田林网建设。坚持创建为民、创建惠民，深入开展群众性精神文明创建活动，全面提升城市公共文明指数和文明城市创建整体水平。

　　三、加速转型，实现开局之年新突破2011年是实施“十二五”规划的开局之年，做好今年的各项工作，意义重大，影响深远。我们将认真贯彻党的十七大和十七届五中全会以及中央、省经济工作会议精神，按照市委六届二十一次全体（扩大）会议的要求，深入贯彻落实科学发展观，大力开展招商引资推进年、项目建设推进年、文明城市创建年、发展环境优化年活动，着力保障和改善民生，努力保持经济平稳较快发展，促进社会和谐稳定。

　　综合考虑各种因素，2011年全市经济社会发展的主要预期目标是：地区生产总值增长12%以上；财政收入增长20%以上；规模以上工业增加值增长18%以上；全社会固定资产投资增长30%以上；社会消费品零售总额增长19%；外贸进出口总额增长20%；城镇居民人均可支配收入增长11%以上；农民人均纯收入增长12%；城镇登记失业率控制在4.2%以内；节能减排完成省政府下达的目标任务；人口自然增长率控制在省下达指标以内。

　　实现上述目标，我们将重点做好五个方面的工作：（一）强力推进大招商，持续增强发展后劲。牢固树立“发展靠投入、投入靠招商”的理念，扎实开展招商引资推进年活动，以招商引资的大突破，实现大投入、大发展。

　　突出招商引资重点。主攻重点产业，成立工业与新兴产业、商贸物流（旅游）产业、食品产业、房地产业、文化教育与创意产业五个产业招商中心，围绕现有基础和优势，谋划一批优质项目。

　　主攻重点企业，加强与央企、知名民企的对接合作，深入研究世界500强、国内500强企业发展战略和投资方向，选准项目，点对点主动上门招商。主攻重点区域，面向苏浙沪、珠三角等地区，组建五个驻外招商分局，驻点招商，捕捉信息，锁定目标，积极主动承接产业转移。全年引进内资370亿元，增长50%以上；引进投资规模3000万元以上项目100个。

　　狠抓招商项目落地。建立招商项目库和客商资源库，梳理跟进意向项目，跟踪落实签约项目，加快推进落地项目，尽快把意向转化为合同，合同转化为开工，开工转化为投产，努力提高项目履约率、资金到位率和投产达产率。

　　对项目进行全过程、全方位跟踪服务，促进项目早建成、早投产、早见效。

　　提升园区承载能力。坚持以规划为引领，持续加大开发区和园区建设开发力度，持续提升园区承载能力，年内每个县区开发区扩区3-5平方公里。按照先规划后建设、先地下后地上、先环境后招商的模式，举全市之力，启动市开发区南区一期10平方公里建设。高起点编制临涣工业园20.4平方公里发展规划，启动园区路网等基础设施建设。

　　继续办好2011中国（淮北）煤矿机械博览会，筹办首届中国（淮北）食品工业博览会。

　　完善招商工作机制。改进招商引资目标任务分解和考核方式，按月调度通报招商引资情况，定期召开招商引资述职大会。改进优惠政策兑现方式，科学合理配置有限资源，探索建立全市招商信息统一平台。推行为企服务全程代理制，实行“六个一”工作机制，提升行政审批、项目验收、政策兑现等环节的服务效率，大力营造亲商、安商、富商的社会氛围。

　　（二）强力推进大投入，加快城市转型步伐。深化投融资体制改革，拓宽融资渠道破解土地、环境等难题，扎实开展项目建设推进年活动，充分发挥投资拉动经济增长的引擎作用。

　　加大工业投入保增长。强力推进94个工业类项目建设，确保完成投资118.3亿元。围绕打造煤电化千亿板块，积极推进邹庄煤矿、大唐淮北发电厂虎山工程、临涣煤焦化二期、粗苯加氢精制、鸿源焦化扩建工程等项目建设。围绕打造黄淮海食品工业高地，加快建设思朗食品、神华食品、顺发食品、康宏园二期等农副产品深加工项目。围绕建设国内知名的煤矿机械制造基地，力促安徽机电装备、富士特铝业、淮工机械、中能矿机等加工制造业项目尽快建成。围绕构建纺织—印染—服装产业链，加快推动顾威制衣、唯一服饰等项目建设，支持华孚色纺、维科印染等骨干企业扩大产业规模。

　　加大服务业投入调结构。强力推进31个服务业类项目建设，确保完成投资30.亿元。加快中泰国际广场、翔宇物流等在建项目进度，开工建设城市广场二期、长通伟业物流、张寨商贸等项目。继续实施“万村千乡市场工程”，新建连锁农资农家店100家，新建标准化菜市场2家。建立多元化金融服务体系，全年引进股份制银行2家，新组建担保公司4家、小额贷款公司5家以上，村镇银行和市农村商业银行年内挂牌营业，濉溪县农村信用社转制组建农商行。力争银行业金融机构全年新增贷款100亿元以上。加速龙脊山旅游开发区建设，办好塔山石榴旅游文化节，提升旅游业发展水平。

　　加大基础设施投入优环境。强力推进41个基础设施类项目建设，确保完成投资47.3亿元。加快建设公路、铁路、水运综合交通体系，继续实施省道202二期一级公路改造，开工建设相山—岳集快速通道、濉永路改建项目，完成泗许高速淮北段征地拆迁及部分路基工程，积极推进符夹线铁路电气化改造，做好淮北—徐州城际铁路前期工作，启动南坪港区和航道建设。实施农村清洁工程，新建示范镇6个、示范村4个。加强市县电网规划建设，启动濉溪变电站扩建工程，开工建设铁佛、滂汪变电站。

　　加大技术创新投入增实力。设立新兴产业发展基金，加强与高等院校和科研院所的联系合作，积极推进新能源、新材料等新兴产业发展和集聚，重点推动金蟾制药、巴迪生物、天象龙盟环保纸项目年内投产，开工建设中国矿业大学科技园淮北孵化基地。支持企业采用先进适用技术改造提升传统产业，实施技改项目240个，完成投资122亿元，新增省级以上技术中心3家、省名牌产品6个。加快推进国家技术创新工程试点工作，组织实施重大科技攻关项目30个，新增国家高新技术企业、创新型试点企业10家。积极推进国家循环经济试点市和循环经济园区建设，加强重点企业、重点行业和关键领域的节能减排工作，确保完成年度目标任务。

　　（三）强力推进大统筹，加速城乡一体化进程。坚持以工补农，以城带乡，市、县区一体，推动城乡统筹协调发展。

　　加快发展现代农业。开展小麦千斤市创建活动，建设100万亩核心示范区，争取5—6个乡镇率先建成小麦高产千斤镇。实施玉米振兴计划，重点建好40万亩高产核心示范区，继续提高玉米单产。以发展城郊型农业为重点，落实蔬菜种植小区奖补政策，实施新一轮“菜篮子”工程，新增设施蔬菜5000亩，创建高效经济作物标准示范园10个。持续扩大规模养殖比重，新建标准化养殖示范小区30个。大力推进农业产业化，重点扶持培育市级以上龙头企业80家。启动农业示范区、农业循环经济园建设。实施王引河治理和澥河洼地治理工程，加快小型农田水利设施建设，新增和改善灌溉面积38万亩、除涝面积16万亩。

　　加速城乡一体化进程。扩大城乡一体化试点范围，新建农民集中居住区20个，集聚人口6万人。建立多元化的资金投入机制，鼓励民间资本参与城乡一体化建设，整合教育、卫生、广电等公共资源，加大道路、给排水、垃圾清运、网络通讯等配套设施建设力度，逐步完善集中居住区功能。把土地整治作为新农村建设、城乡统筹的主抓手，作为政府“一把手”工程强力推进，全年整治土地2.6万亩。落实“扩权强镇”试点工作，支持县区加快发展，支持濉溪县尽快进入全省十强县。

　　创新农村发展体制机制。规范农村土地流转，落实农村土地流转合同制度，建立价格指导机制及跟踪检查制度，力争全年新增规模土地流转面积2万亩。加强农业技术推广、动物防疫、农业信息化服务、农产品质量安全体系建设，引进示范推广新品种10个、新技术10项。推进农民专业合作、农村土地合作、农村金融互助合作、农村社区合作，重点培育40家规范化示范合作组织。

　　做好各项农业政策性保险工作，降低农业生产风险。

　　（四）强力推进大建设，奋力争创全国文明城市。扎实开展文明城市创建年活动，以城市大建设推动城市功能品位的大提升，力争跨入全国文明城市行列。

　　启动东部新区建设。完善新区路网，确保宿丁路等12条道路以及梧桐路北段改造年底前建成通车，加快淮海东路、长山南路南段等道路建设。启动政务新区建设，加快建设市委党校、淮北矿业集团和淮海集团公司办公新区、公安监管中心，开工建设淮北师范大学新校区，做好淮北职业技术学院新校区、市人民医院东院区前期工作。高标准建设东部新区污水管网，配套建设城市绿地、公交站场、天然气加气子站等公用设施。

　　加强生态环境保护。完成采煤沉陷区水资源利用与湿地修复二期主体工程，加强南湖、东湖、中湖、相湖等景区水体整治。实施矿山地质环境重点项目一期工程，继续推进东部荒山绿化，加快相山公园改扩建，改造南湖湿地公园，建设时代公园“清风苑”，建成桓谭公园。开展群众健康环保专项行动，清理和整治违法排污企业，完善城乡垃圾收集系统，加大畜禽养殖和农村面源污染防治。

　　增强城市综合功能。提升淮海路、孟山路特色商业街档次，积极推进东部新城区商业综合体建设。完善老城区供气、供水、排水管网，加快城市中水回用项目、龙湖工业园污水处理厂配套管网工程、烈山污水处理厂建设。实施以硬化道路、美化亮化环境为主要内容的旧住宅区综合整治，投资2亿元，改造60个旧住宅小区，以此作为文明创建突破口，作为为民创建的一件实事抓细抓好。

　　强化城市规划管理。充分发挥规划的龙头作用，推动市县规划一体化，科学编制《淮北市城市近期建设规划（2011—2015年）》。开展以城带乡、矿镇共建结对帮扶活动，完成“数字化”城市管理监控中心二期工程，建立执法管理预防机制。抓好背街小巷、农贸市场等重点部位的环境整治，依法查处违章建筑。继续开展“千名干部下基层、同创共建文明城”活动，全面做好第三届全国文明城市测评迎检工作。

　　（五）强力推进民生大改善，加快构建和谐淮北。坚持把保障和改善民生作为政府工作的出发点和落脚点，切实解决好事关群众切身利益的突出问题。

　　着力改善民计民生。优化财政支出结构，新增财力优先向民生倾斜，组织实施好33项民生工程。积极推进国家级创业型城市建设，完善创业政策，健全创业机制，优化创业环境，鼓励百姓创家业、能人创企业、回乡创新业。落实更加积极的就业政策，认真做好高校毕业生、城镇就业困难人员和农民工就业工作。全年新增就业岗位3.万个，实现下岗失业人员再就业7100人，新转移农村劳动力8000人。提高农村五保户供养、重度残疾人生活和农村义务教育阶段生均公共经费补助标准，扩大高校、中职学校家庭困难学生资助范围，实施普通高中家庭经济困难学生资助。

　　抓好新型农村社会养老保险试点、养老保险省级统筹工作，确保符合参保条件的失地农民应保尽保。完善新型农村合作医疗制度。提高优抚对象、城乡低保、医疗救助水平，健全城乡临时救助制度。加快农村五保供养服务机构建设，力争集中供养率达到35%以上。实施旧城改造项目38个；新建廉租住房3400套；投资7000万元，完成相山风景区安置房建设。整顿和规范市场秩序，加强市场运行和物价监测调控，保持市场物价基本稳定。

　　大力发展社会事业。坚持优先发展教育，推进义务教育均衡发展，建成市实验高中新校区，完成校舍安全工程，加快中小学校标准化建设。大力发展职业教育，财政对市域内农村学生进入职业学校就读生给予资助，加快培养和储备更多实用人才。加强文化建设，建成市广电中心，实施乡镇综合文化站、农家书屋、广播电视“村村通”工程，开展第三次全国文物普查，做好隋唐大运河保护和申遗工作。加快发展公共卫生事业，建立市级公共卫生应急指挥平台，抓好市中医院二期等项目建设。大力开展全民健身运动，举办全市第九届运动会。加强人口和计划生育工作，综合治理出生人口性别比，做好流动人口计生管理与服务。完成第六次全国人口普查。关注残疾人事业发展，创建全国无障碍建设城市。全面加强信息化条件下的保密工作。抓好机关事务、档案、地方志工作，推进外事侨务、对台、贸易促进、地震、气象、红十字会工作。重视和推动妇女儿童、老龄、社会福利、慈善等事业发展，支持工会、共青团、妇联等人民团体参与社会管理和公共服务。

　　全力维护社会和谐稳定。进一步健全政府应急管理机制，加强应急救援队伍建设，提高保障公共安全和处置突发事件的能力。强化安全生产责任制，深入排查和消除事故隐患，大力开展交通、消防和建筑安全等专项整治，突出抓好煤矿、非煤矿山、交通运输、危险化学品、烟花爆竹、高层住宅等重点领域的安全监管，严防各类安全生产事故发生。加强工商行政管理和质量技术监督工作，实施食品药品放心工程，做好防汛抗旱和救灾减灾工作。进一步加强社会治安综合治理，加快数字化防控体系建设，扎实开展法制宣传和法律援助，严密防范、依法打击各种违法犯罪活动，努力为人民群众创造安全、安定、有序的工作生活环境。加强和改进新形势下的信访工作，畅通民意表达渠道。认真做好民族宗教工作。加强国防动员和人民防空建设，支持驻淮部队建设，提高双拥优抚安置工作水平，巩固发展军政军民团结。

　　四、服务转型，推动政府效能新提升  面对发展的重任，肩负人民的重托，我们将倍加珍惜转型发展的宝贵机遇，担当起奋力崛起的历史使命，聚精会神搞建设，一心一意谋发展，以发展的新业绩取信于民。

　　（一）坚持执政为民，提升群众工作能力。只有始终与人民群众同呼吸、共命运、心连心，我们的事业才有根基，我们的发展才有动力。大力弘扬密切联系群众之风，做到思想上尊重群众、感情上贴近群众、工作上依靠群众、生活上关心群众，虚心向群众学习、热心为群众服务、诚心接受群众监督。始终坚持群众利益无小事，切实维护好、实现好、发展好人民群众的根本利益，认真解决群众反映强烈的突出问题，做到情为民所系、权为民所用、利为民所谋，努力营造心齐劲足、团结奋进的良好局面。

　　（二）坚持解放思想，提升科学发展能力。解放思想，永无止境，与时俱进，永恒规律。面对新形势、新挑战，只有推进思想大解放、真解放，才能实现大跨越、大发展。继续开展“创先争优”活动，积极打造学习型城市，推进知识的大更新、思维方式的大转变、工作方式方法的大创新，坚决破除小富即安、得过且过的小农意识，坚决破除等、靠、要的思想倾向，坚决破除坐而论道、评头论足的不良风气，敢想敢干，快干实干，用改革发展的实绩检验解放思想的成果，真正把解放思想的成效体现到科学发展、转型发展上来。

　　（三）坚持依法行政，提升执行落实能力。自觉接受人大和政协监督，认真办理人大代表建议、议案和政协委员提案，广泛听取各民主党派、工商联、无党派人士和工青妇等人民团体的意见，高度重视人民群众与新闻媒体舆论监督。坚持科学民主决策，深入推进政务公开，让权力在阳光下运行。扎实开展发展环境优化年活动，真抓实干，勇挑重担，奋发进取，知难而进，努力做到时时在状态、天天有激情，各项工作皖北争一流、全省争上游。

　　（四）坚持反腐倡廉，提升廉洁从政能力。牢记“两个务必”，大力精简会议、文件和事务性活动，压缩一般性财政支出，深化部门预算和财政支出绩效评价改革，努力降低行政成本，把有限的财力用到经济建设上来，用到增添发展后劲上来，用到保障和改善民生上来。注重惩防并举，落实“一岗双责”，扎实推进反腐倡廉建设，严肃查处违法违纪行为，坚决纠正损害群众利益的不正之风，努力打造为民、务实、清廉、高效政府。

　　各位代表！奋起直追、奋力崛起是全市广大干部群众的迫切要求，加快转型、加快发展是我们义不容辞的神圣使命。让我们在中共淮北市委的坚强领导下，紧紧依靠和团结全市人民，凝心聚力，开拓进取，为圆满完成“十二五”和全年的各项目标任务，奋力争当皖北崛起排头兵而努力奋斗！
